# Alpha lipoic acid inhibits oxidative stress‐induced apoptosis by modulating of Nrf2 signalling pathway after traumatic brain injury

**DOI:** 10.1111/jcmm.14296

**Published:** 2019-04-15

**Authors:** Dayong Xia, Xiaofu Zhai, Honglian Wang, Zhiyong Chen, Chuanjing Fu, Meihua Zhu

**Affiliations:** ^1^ Department of Neurosurgery The First Affiliated Hospital of Wannan Medical College Wuhu Anhui Province China; ^2^ Department of Neurosurgery Huai’an Second People’s Hospital, Xuzhou Medical College Huai’an Jiangsu Province China; ^3^ Department of Radiology Huai’an Fourth people’s Hospital Huai’an Jiangsu Province China; ^4^ Department of Anesthesiology the Second Affiliated Hospital of Nanjing University of Chinese Medicine Nanjing Jiangsu Province China; ^5^ Department of Neurosurgery Jiangsu Hospital of Traditional Chinese Medicine Nanjing Jiangsu Province China

**Keywords:** ALA, neuronal apoptosis, Nrf2, traumatic brain injury

## Abstract

Alpha lipoic acid (ALA) is a powerful antioxidant which has been widely used in the treatment of different system diseases, such as cardiovascular and cerebrovascular diseases. But, there are few studies that refer to protective effects and potential mechanisms on traumatic brain injury (TBI). This study was carried out to investigate the neuroprotective effect following TBI and illuminate the underlying mechanism. Weight drop‐injured model in rats was induced by weight‐drop. ALA was administrated via intraperitoneal injection after TBI. Neurologic scores were examined following several tests. Neurological score was performed to measure behavioural outcomes. Nissl staining and TUNEL were performed to evaluate the neuronal apoptosis. Western blotting was engaged to analyse the protein content of the Nuclear factor erythroid 2‐related factor 2 (Nrf2) and its downstream protein factors, including hemeoxygenase‐1 (HO‐1) and quinine oxidoreductase‐1 (NQO1). ALA treatment alleviated TBI‐induced neuron cell apoptosis and improved neurobehavioural function by up‐regulation of Nrf2 expression and its downstream protein factors after TBI. This study presents new perspective of the mechanisms responsible for the neuronal apoptosis of ALA, with possible involvement of Nrf2 pathway.

## 1 INTRODUCTION

Traumatic brain injury (TBI) is a public health problem in modern society.^1^ TBI causes primary mechanical injury of neuron cells and initiates secondary damage that occur after the primary damage. Many investigations indicated that secondary damage result in neuronal apoptosis is the main reason of poor outcome after TBI which is progressive from hours to days.^2^ Many pathological process were reported to be related to the neuronal apoptosis in the secondary damage.^3^, ^4^ So, relieve of neuronal apoptosis was the fundamental therapy purpose of TBI. However, despite long‐sustained efforts have been made to expound its mechanisms, its effective treatment is still not raised.

Alpha‐lipoic acid (ALA) is a kind of naturally occurring compound which is reported to have antioxidant activities.^5^ It is also a powerful antioxidant inhibiting ROS‐induced damaging effects. Previous study has shown that ALA exerts neuroprotective effects against TBI by suppressing the mitochondrial apoptotic pathway.^6^, ^7^ In addition, ALA was found to have the function to regulating the level of anti‐oxidative enzymes as well as preventing hepatic inflammatory responses and oxidative stress after trauma.^8^ However, the effects of ALA to TBI have not been investigated.^9^ However, the mechanisms of the protective function of ALA and its effects on apoptotic mechanisms after TBI remain unknown.

Many researches have proved that nuclear factor erythroid 2‐related factor 2 (Nrf2), is a key leucine zipper redox sensitive transcription factor which has been reported to be an regulator in cell antioxidant mechanism and considered to be a protector for many organs in different models.^10^, ^11^ Under normal conditions, Nrf2 is sequestered in the cytoplasm where it is interacted with the Kelch‐like ECH associating protein (Keap1).^12^ The enzymes regarded as antioxidants including superoxide dismutase (SOD) and glutathione peroxidase (GPx). All of these enzymes form a powerful antioxidant defense mechanism. In conditions of stimulations, Nrf2 translocates from the cytoplasm to the nucleus, and binds to the antioxidant response element (ARE) which is a regulatory element, and resulting in protective response including many antioxidant enzymes up‐regulation, such as quinine oxidoreductase‐1 (NQO1) and heme oxygenase‐1(HO‐1).^13^, ^14^ Except that, it has been reported that it is an important regulator in protect TBI‐induced second brain injury.^15^ It is reasonably believed that the activation of Nrf2 could be involved in the post‐TBI neuroprotective effect of ALA administration. In this study, it is evaluated the influence of ALA on the cerebral up‐regulation of Nrf2 activity after TBI.

## 2 MATERIALS AND METHODS

### 2.1 Experimental animals

Male Sprague‐Dawley rats (weight: 250‐280 g) were gained from experimental animal center of the Second Affiliated Hospital of Nanjing University of Chinese Medicine. Animal experiments were approved by the Animal Care and Use Committee in according to the rules for animal research. The rats were raised under controlled temperature (24 ± 0.5℃) and free access to forage and drinking water was allowed for 7 days.

### 2.2 Model of TBI

Rats were placed in a stereotaxic frame after intraperitoneal anaesthetized by 10% chloral hydrate (400 mg/kg). About 2‐cm midline scalp incision was made to expose the skull. Then, a 6‐mm hole was performed over the left parietal cortex; the centre of the hole was 2.5 mm lateral to the midline on the mid‐coronal plane. During the operation, the dura should not be broken. After that, a 40‐g weight was released onto the dura from 25 cm high.^16^ The scalp wound was sutured after that. Sham animals received the same procedures except weight injured.

### 2.3 Experimental groups and drug administration

Part 1: Thirty‐five rats were used for this experiment. After operation, five of them died and were excluded. The remaining 30 rats post‐TBI were divided into five groups(n = 6): Sham group; Sham + ALA group; TBI group; TBI + vehicle group; TBI + ALA group for the neurological severity score (NSS) system at 1, 2, 3 and 7 days after TBI.

Part 2: To determine the role of Nrf2 after TBI, rats (137 rats were used, 17 rats died) were divided into four groups (n = 24 each): Sham group; Sham + ALA; TBI group; TBI + vehicle group; TBI + ALA group; All the animals were executed at 24 hours following TBI. Evaluation item in this stage contain, Real‐Time Quantitative Polymerase Chain Reaction, Western blot, Immunohistochemical staining, Nissl staining, TUNEL, Malondialdehyde content, Superoxide dismutase and Glutathione peroxidase activity.

ALA (T5625; Sigma‐Aldrich, MO, USA) was dissolved in the vehicle solution [10% dimethyl sulfoxide (DMSO) in corn oil]. Rats in the TBI + ALA and TBI + vehicle groups were administrated (i. p) ALA (100 mg/kg body weight) or equal volumes of DMSO, respectively, 30 minutes after TBI. The dose of drug was confirmed according to previous study.^16^

### 2.4 Tissue preparation

Rats were intraperitoneally anaesthetized by sodium pentobarbital (50 mg/kg; i.p) and perfused intracardially with cold (4°C) 0.9% saline by thoracotomy towards the cannula. The left cortex peri‐contusion was rapidly removed and ipsilateral cerebral cortex tissue 3 mm from the margin of the contusion site was frozen in liquid nitrogen, and then preserved at −80°C freezer. The tissues of remaining rats were immersed in 4% paraformaldehyde, kept at −80°C overnight.

### 2.5 Neurobehavioural assessment

Severity of neurological deficit was evaluated at 24 hours by the neurological severity score (NSS) system. It is graded on a scale of 0‐18(0 = minimum deficit, and 18 = maximum deficit). NSS scoring roots on motor, sense reflex, and balance functions. Thus, the higher score means the greater injury. All the animals were recorded at 1, 2, 3 and 7 days after TBI. The behavioural test was carried out by two independent investigators who were blind to the experimental groups.

### 2.6 Real‐time quantitative polymerase chain reaction

Total RNA was extracted from harvested cortex using RNAiso Plus (TaKaRa Bio, Dalian, China). Spectrophotometer and 1% agarose gel electrophoresis were used to detect the concentration of total RNA. Portion of RNA was reverse transcribed to cDNA with the Prime Script RT reagent kit to avoid RNA degradation. The primer sequences were designed as follows: NQO1: F: 5′‐CAT TCT GAA AGG CTG GTT TGA‐3′; R:5′‐CTA GCT TTG ATC TGG TTG TCAG‐3′; HO‐1: F: 5′‐ATC GTG CTC GCA TGA ACA CT‐3′; R: 5′‐CCA ACA CTG CAT TTA CAT GGC‐3′; β‐actin: F: 5′‐AGT GTG ACG TTG ACA TCC GTA‐3′; R: 5′‐GCC AGA GCA GTA ATC TCC TTCT‐3′. The PCR analysis was performed by the Mx3000P System (Strata gene, San Diego, CA, USA).

### 2.7 Western blot analysis

Protein extracts and diluted in sodium dodecyl sulphate loading buffer were electrophoresed on 10% or 15% sodium dodecyl sulfate‐polyacrylamide gels and transferred to polyvinylidene fluoride membranes (Bio‐Rad Lab, Hercules, CA, United States). The membranes were blocked with 5% non‐fat milk for 2 hours, and then incubated overnight at 4°C with Nrf2 (1:1000, Abcam, Cambridge, MA, USA), Bcl‐2 (1:200, Santa Cruz, CA, USA), Cleaved caspase‐3 (1:1000, Cell Signaling, Danvers, MA, USA), Bax (1:200, Santa Cruz, CA, USA), histone H3 (1:1000, Cell Signaling Technology, Beverly, MA, USA), Uncleaved caspase‐3 (1:400, Cell Signaling, Danvers, MA, USA), HO‐1 (1:200, Santa Cruz Biotecnology), NQO1 (1:1000, Abcam, Cambridge, MA, USA), β‐actin (1:5000, Bioworld Technology, MN, USA). After being washed with TBST for three times (15 minutes each), the membranes were subsequently incubated in the secondary antibody (1:1000, Bioworld Technology, MN, USA) at room temperature for 2 hours. The protein bands were exposed to Tanon‐5200 Chemiluminescent Imaging System and strip grey levels were quantified by software (Bio‐Rad Laboratories, Inc).

### 2.8 Immunohistochemical staining

Brain tissue samples were cut at 5 μm after fixing in formalin for 3 days. Sections were washed for 15 minutes in phosphate‐buffered saline (PBS), and were immersed in 10% goat serum containing PBS‐Triton (0.3%) for 1 hour at room temperature. After that, the sections were incubated overnight at 4°C with primary antibody against Nrf2 (1:400, Santa Cruz, CA, USA). After three washes with PBS, counterstain was done by diaminobenzidine and haematoxylin. Of course, control tissue was performed without the primary antibody step.

### 2.9 Terminal deoxynucleotidyl transferase‐mediated dUTP nick‐end labelling analysis

The apoptotic cells were conducted by using a TUNEL detection kit (Roche Inc, Indianapolis, USA). The sections were incubated at 37°C with TUNEL reaction fluid for 60 minutes add the digoxigenin‐conjugated dUTP to the 3′‐OH ends of fragmented DNA and washed with PBS three times for 45 minutes followed by blocking with 10% goat serum in 0.1 m Tris for 15 minutes. The slides were covered by microscopic glass with mounting medium after three washes again. DNA was fixed by streptavidin‐ HRP peroxidase (1:40 dilution) and dyeing with DAB Chromogen. The apoptotic cells showed cell atrophy with crimp brown nucleus was performed by ZEISS HB050 inverted microscope. The positive cells were monitored and analysed by two observers blinding to the experiment. TUNEL‐positive cells in four aspects from each sample were elected for quantification. The average percentage of these aspects was confirmed as the ultimately data.

### 2.10 Malondialdehyde content, Superoxide Dismutase and glutathione peroxidase activity

The MDA content, SOD and GPx activity were measured with a spectrophotometer using the appropriate kits (Nanjing Jiancheng Biochemistry Co, Nanjing, China) following the manufacturer's instructions. Total protein concentration was determined by the Bradford method. The MDA level, SOD and GPx activity was expressed as nmol/mg protein and U/mg protein, respectively.

### 2.11 Statistical analysis

All statistical analyses were performed by SPSS, version 17.0 (SPSS, Inc, Chicago, IL, USA). Data are expressed as the mean ± SD and the differences were analysed using ANOVA and Tukey tests. Statistical significance was set at value of *P* < 0.05.

## 3 RESULTS

### 3.1 ALA improved neurological function after TBI

**Figure 1:**
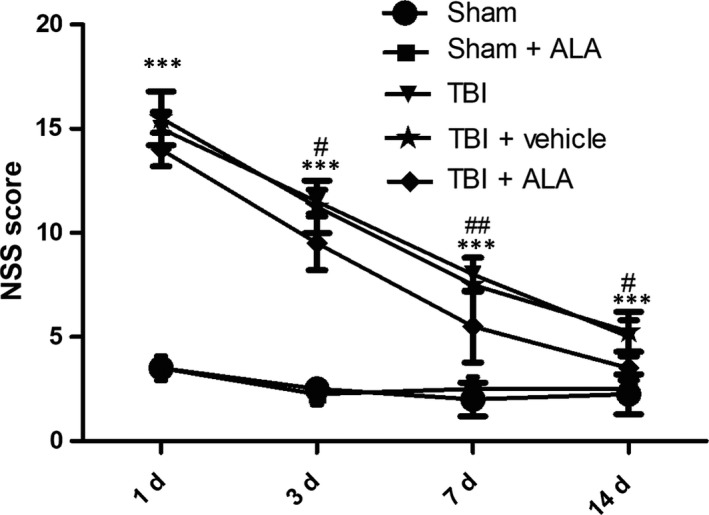
Administration of ALA promotes neurobehaviour after TBI. Higher neurological severity scores (NSS) reflect more severe injury. Data are expressed as the mean ± SD. ***P < 0.001, versus sham group; ^#^ *P* < 0.05, ^##^ *P* < 0.01, versus TB + vehicle group

Animals were trained through the test for 3 days before experiment. NSS score was evaluated at 1, 2, 3 and 7 days post‐TBI (Figure 1). NSS score was higher in the TBI and TBI + vehicle group than the sham and sham + ALA group after 1 day after TBI. However, at 2, 3 and 7 days following TBI, NSS score declined in the TBI + ALA group compared with the TBI and TBI + vehicle group.

### 3.2 ALA suppressed neuronal apoptosis after TBI

**Figure 2:**
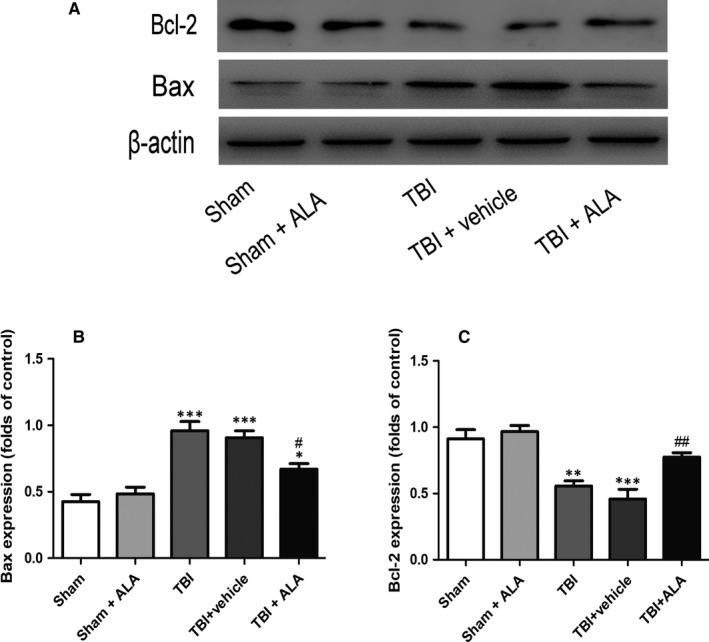
The effect of ALA on cleaved caspase‐3, Bax, and Bcl‐2 expression in cortical neural cells in rat model of TBI was assessed by Western blot analysis. Expression was normalized to the level of β‐actin. Data are presented as mean ± SD. *P < 0.05, **P < 0.01, ***P < 0.001 versus sham group; ^#^ *P* < 0.05, ^##^ *P* < 0.01 versus TBI + vehicle group

As shown in Figure 2, the cell number counting showed that few positive cells were found in the sham group. Furthermore, the apoptotic index was raised after TBI insult. There was no difference between the TBI and the vehicle‐treated group; however, lower level of apoptotic cells was found in the TBI + ALA group.

As shown in Figure 2A, expression of cleaved caspase‐3 significantly increased in the TBI group and TBI + vehicle group compared with the sham and sham + ALA group, and it was notably suppressed after ALA administration. Except that, the level of cleaved caspase‐3 was significantly rose following TBI and notably down‐regulated after ALA administration.

### 3.3 ALA reduced the expression of apoptotic factors

The protective effects of ALA against TBI‐induced neural apoptosis were examined by Western blot analysis (Figure 2). These effects were reversed in TBI rat treated with ALA that the expression of Bax was inhibited. The expression of the pro‐apoptotic factor Bax increased following TBI when compared with the Sham group (Figure 2A), whereas the expression of the anti‐apoptotic factor Bcl‐2 decreased when compared with the Sham group (Figure 2A, B). However, treatment with 100 mg/kg of ALA reversed the expression levels of Bax and Bcl‐2 relative to the TBI + vehicle group.

### 3.4 ALA promoted the expression of Nrf2

**Figure 3:**
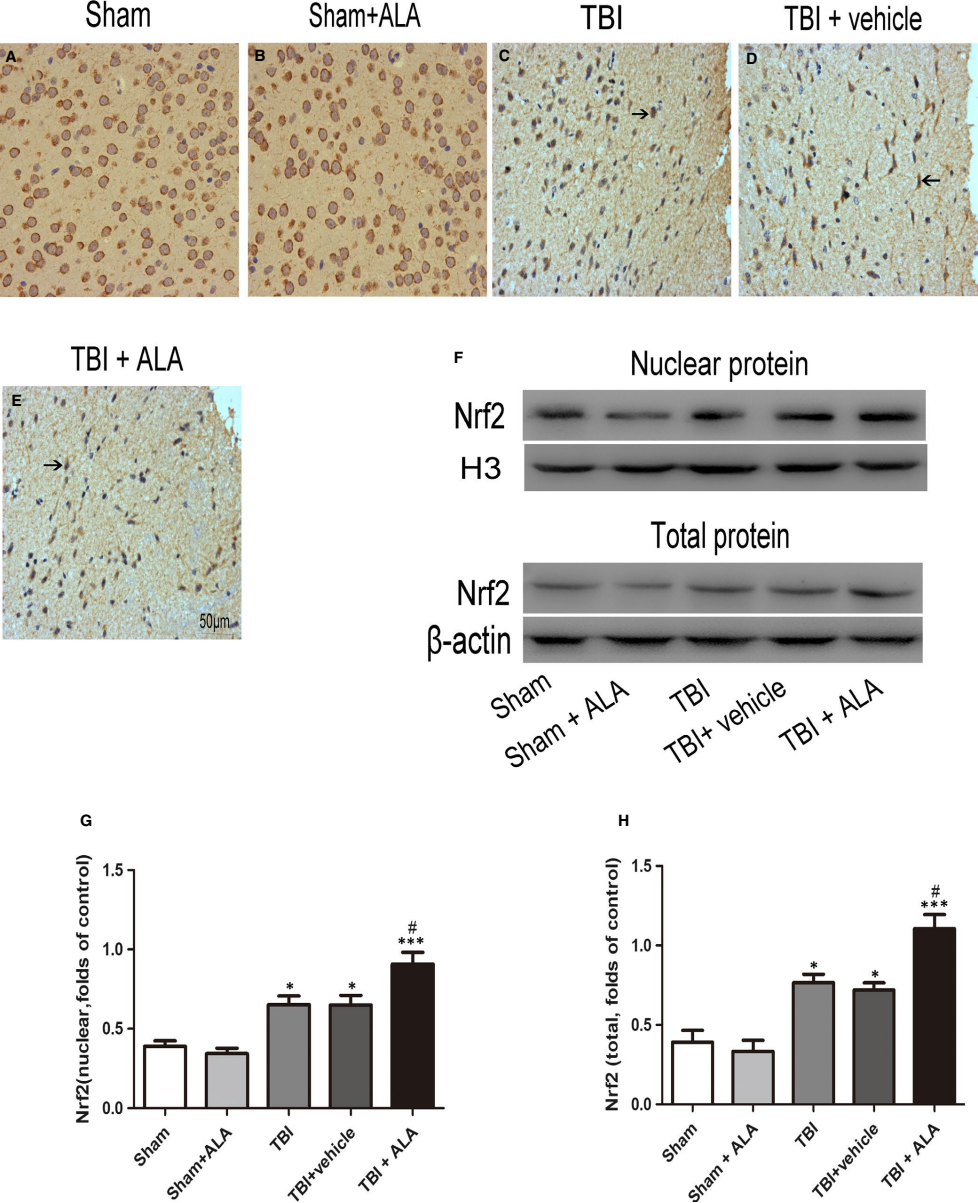
Alpha lipoic acid promoted translocation of Nrf2 from cytoplasm to nucleus and enhanced Nrf2 binding. A, The representative photomicrographs showing Nrf2 immunohistochemistry of tissue from different group after TBI. (B, C) The total and nuclear Nrf2 expression after ALA treatment in rat with TBI was measured by Western blot. Bars represent the mean ± SD. *P < 0.05, ***P < 0.001 compared with the sham group; ^#^ *P* < 0.05, ^##^ *P* < 0.01 versus TBI + vehicle group. Black arrows: Nrf2 positive neuron cell

Western blot analysis and immunohistochemistry were applied to detect the distribution and level of Nrf2. Subsequently, Nrf2 was mainly located in the cytoplasm in the sham and sham + ALA (Figure 3A, C). Meanwhile, the expression bosting of Nrf2 was observed in TBI and TBI + vehicle group, whereas the ALA administration showed obvious enhancement in the distribution. Ratio of positive cells were detected a few positive neurons in the Sham group, and, the number of positive cell was up‐regulated following TBI insult (Figure 3A‐C). However, ALA administration increased the percentage of positive cell compared with TBI + vehicle group.

### 3.5 ALA promoted the expression of Nrf2 downstream factors

**Figure 4:**
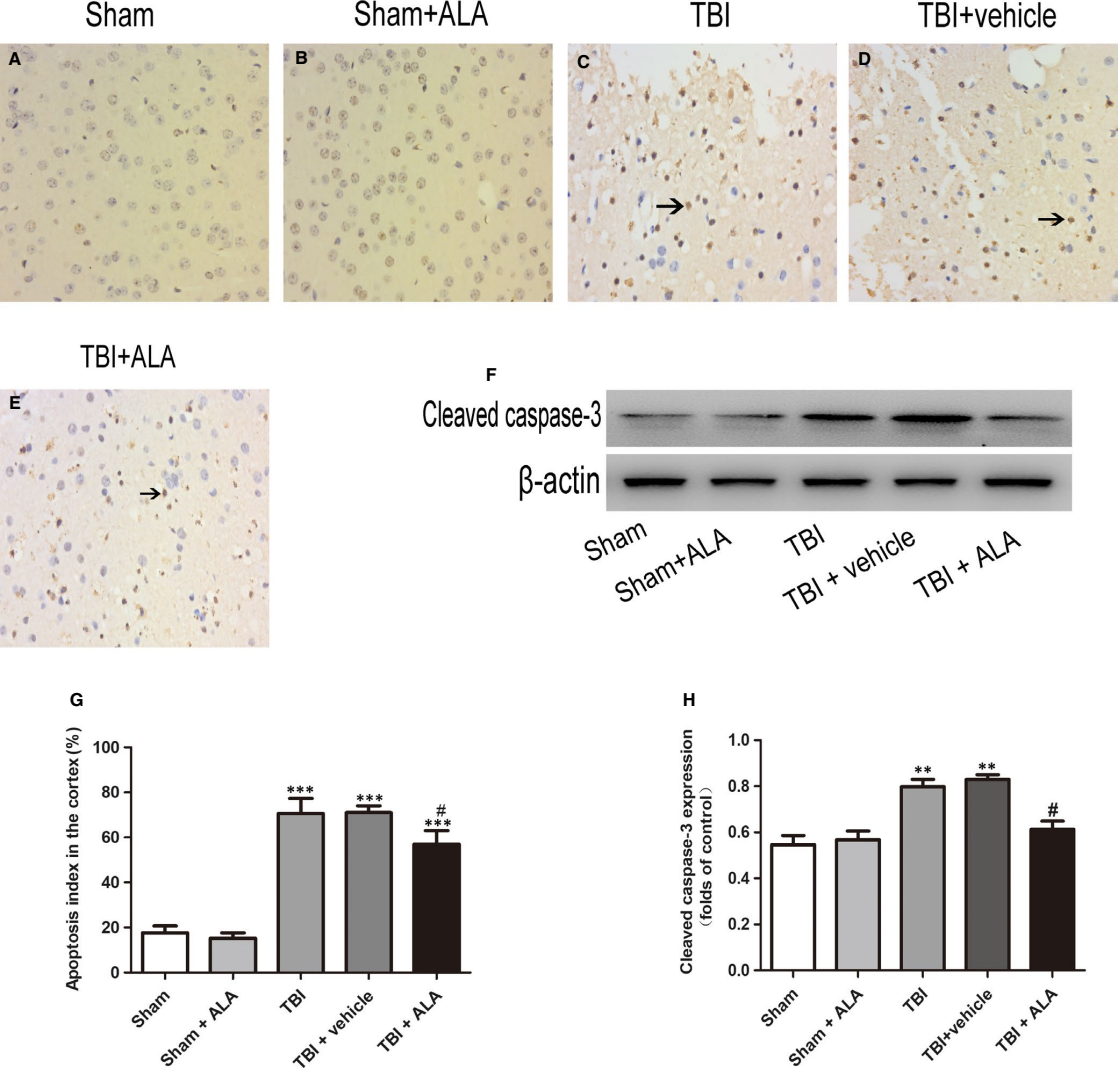
Apoptotic index was determined using TUNEL assays after TBI. ALA treatment significantly decreased the percentage of apoptotic cells after TBI. Data are presented as mean ± SD; ***P < 0.001 versus sham group; ^#^ *P* < 0.05 versus TBI + vehicle group. Black arrows: Nrf2 positive neuron cell

**Figure 5:**
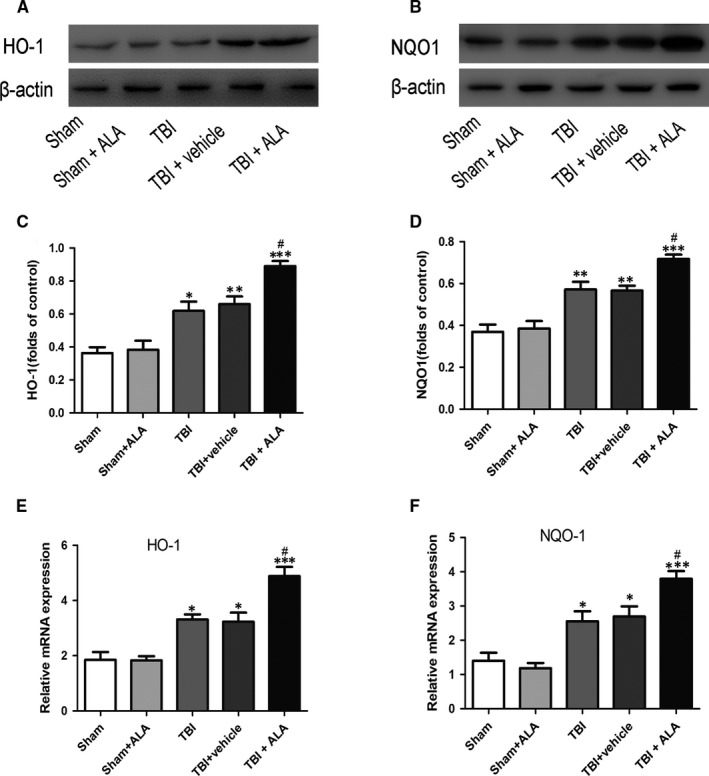
Alpha lipoic acid up‐regulate the expression of Nrf2 downstreams on both protein and mRNA levels. (A, B) Both HO‐1 and NQO1 proteins were up‐regulated after TBI, besides, ALA further elevated their expression in the brain tissue post ALA administration. (C) HO‐1 mRNA was increased after TBI and was further increased with the administration of ALA. Similarly, NQO1 mRNA was elevated after TBI and was further raised with administration of ALA. β‐actin was used as a loading control. Data are presented as mean ± SD, n = 6 per group; *P < 0.05, **P < 0.01 and ***P < 0.001 versus sham group; ^#^ *P* < 0.05 versus TBI + vehicle group

The expression of NQO‐1 and HO‐1 was also measured by Western blot analysis. As shown in Figure 4, NQO‐1 and HO‐1 increased significantly following TBI (Figure 5). ALA treatment further enhanced the level of protein compared with the TBI + vehicle group. These results implied that ALA induced Nrf2 downstream factors expression (Figure 5A,B). Moreover, the result of HO‐1 and NQO‐1 in Western blot analysis was consistent with the mRNA level (Figure 5C).

### 3.6 ALA reduced oxidative stress following TBI

**Figure 6:**
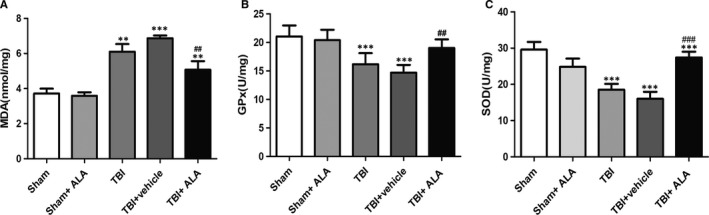
Alpha lipoic acid attenuated oxidative stress caused by TBI. A, Measurements of MDA levels. (B, C) The activity of GPx and SOD. Data represent the mean ± SD. *P < 0.05, **P < 0.01 and ***P < 0.001 versus sham group; ^#^ *P* < 0.05, ^##^ *P* < 0.01, ^###^ *P* < 0.001 versus TBI + vehicle

To evaluate the effect of ALA on oxidative stress induced by TBI, MDA level, SOD and GPx activity, indicators of lipid peroxidation and antioxidant levels, respectively, were assessed. MDA level was increased in the TBI + vehicle group compared with the Sham group. This effect was mitigated by the administration of ALA (Figure 6). GPx and SOD activity were both decreased after TBI, while ALA treatment increased their activity (Figure 6).

## 4 DISCUSSION

In this study, ALA was elected to treat TBI and found improved neurological function. Meanwhile, neuronal apoptosis was decreased along with the up‐regulation of bcl‐2 by ALA administration. ALA is a favourable safety profile in many animal experiment and clinical trials which has the effect of antioxidant and anti‐inflammatory. 17, 18 This finding showed that ALA significantly abated the percentage of apoptotic cells as well as the level of active caspase‐3, which were enhanced by brain injury. Previous study has found that ALA has protective effect against ischaemia/reperfusion injury via regulation of the expression of the bcl‐2.^19^ More important is that ALA administered alleviated the mitochondrial apoptotic pathway following TBI and made the prognosis improve significantly.^20^ Therefore, it was reasonably believed that ALA can alleviate oxidative stress‐induced apoptosis following TBI.^21^ However, the mechanism of it on brain injury has not yet been undefined. In this study, ALA significantly improved post‐TBI neurobehaviour at 2, 3 and 7 days. This involves the anti‐apoptotic Bcl‐2 family member Bax which is confined in the mitochondrial outer membrane which leading to degradation of DNA, terminates in cell apoptosis. Here it was indicated that the level of Bax increased after TBI, imply that the up‐regulation of cleaved caspase‐3, leading to neuronal apoptosis. These results illustrated that ALA could effectively reduce neuron apoptosis.

This beneficial role of ALA led us to consider the neuroprotective effects and the mechanisms underlying oxidative insult.^22^, ^23^ The ability of ALA to stimulate the activity of antioxidant enzymes plays a critical role in its antioxidative characteristics. Lipid peroxidation, which refers to the oxidative degradation of lipids, increases membrane permeability, leading to cell damage. MDA has been utilized as an index of lipid peroxidation. In addition, both of SOD and GPx are antioxidant enzymes, which catalyse the reduction in glutathione.^24^ The conversion of MDA and the activity of SOD and GPx in the cortex of mice with TBI indicate that oxidative stress occurs following TBI.^25^, ^26^ Apart from that, the results of this study also showed that treatment with ALA partly relieves this impact, suggesting that ALA could attenuate TBI‐induced oxidative stress.

To confirm the Nrf2 signal pathway is involved in the neuroprotective role of ALA, the expression of Nrf2 protein after ALA administration was investigated. Previous evidence indicated it plays key role in cellular adaptation to oxidative stress, by elevating phase Ⅱ enzymes such as NQO1 and HO‐1 which were activated after TBI.^27^, ^28^ This study demonstrated that the treatment of ALA resulted in a significant increase in Nrf2 expression, as well as in the level of NQO1 and HO‐1. Additional, the changes of Nrf2 signalling pathway after ALA administration was investigated. This study showed that the migration of Nrf2 from the cytoplasm to the nucleus following TBI insult, and ALA promoted this process. Except that, in this study, total Nrf2 protein was significantly up‐regulated following TBI and was much higher after ALA administration. These findings expound that the ALA influence of Nrf2 not only on its protein expression but also its translocation. All these results suggest that Nrf2 plays an important role in ALA treating craniocerebral injury.

In conclusion, this study demonstrated that ALA may have a protective effect against oxidative reaction induced neural apoptosis following TBI and this effect was likely to associate with the activation of Nrf2 signalling pathway. This study is contributed to illuminate the neuroprotective effect of ALA after TBI and its relation molecular mechanism. All results provide experimental evidence for ALA in the treatment of neurotrauma. There are two limitations in this study. First, ALA was used to elucidate the mechanism of anti‐oxidative stress‐induced apoptosis after TBI. Further studies are needed to confirm the results with primary microglia. Second, this study is an in vitro model, and the optimum concentrations of ALA in vivo remains unknown. Therefore, further investigations are warranted for the elucidation of anti‐ apoptosis after TBI mechanism of ALA in vivo.

## CONFLICT OF INTEREST

The authors have declared that no potential conflicts of interests exist.
