# Corrigendum

**DOI:** 10.1111/jcmm.17547

**Published:** 2022-11-07

In Dayong Xia et al.,^1^ the published article contains errors in Figures 3A and 4F. The correct figures are shown below. The authors confirm all results, and conclusions of this article remain unchanged.

**FIGURE 3:**
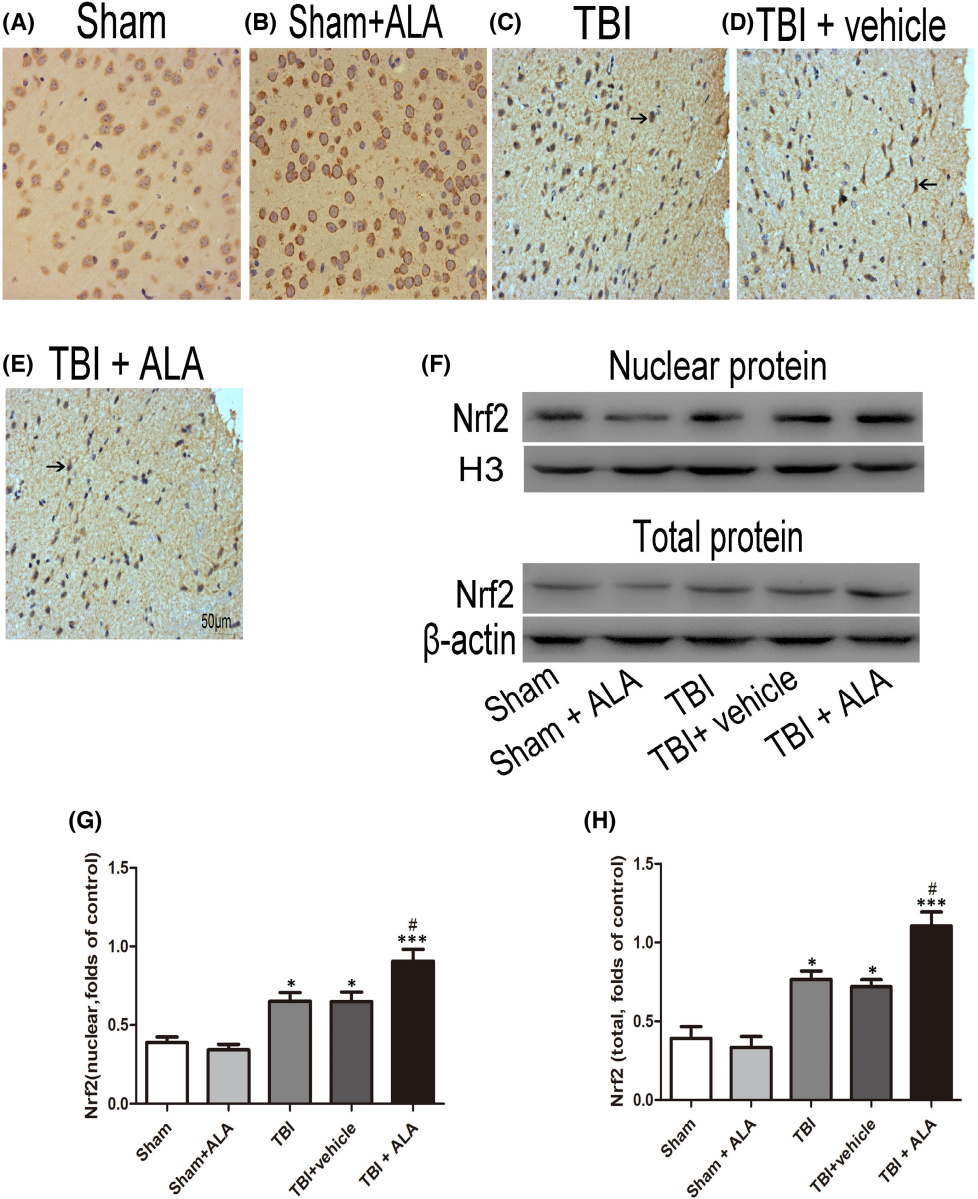
Alpha lipoic acid promoted translocation of Nrf2 from cytoplasm to nucleus and enhanced Nrf2 binding. (A) The representative photomicrographs showing Nrf2 immunohistochemistry of tissue from different group after TBI. (B, C) The total and nuclear Nrf2 expression after ALA treatment in rat with TBI was measured by Western blot. Bars represent the mean ± SD. *p < 0.05, ***p < 0.001 compared with the sham group; ^#^ *p* < 0.05, ^##^ *p* < 0.01 versus TBI + vehicle group. Black arrows: Nrf2‐positive neuron cell

**FIGURE 4:**
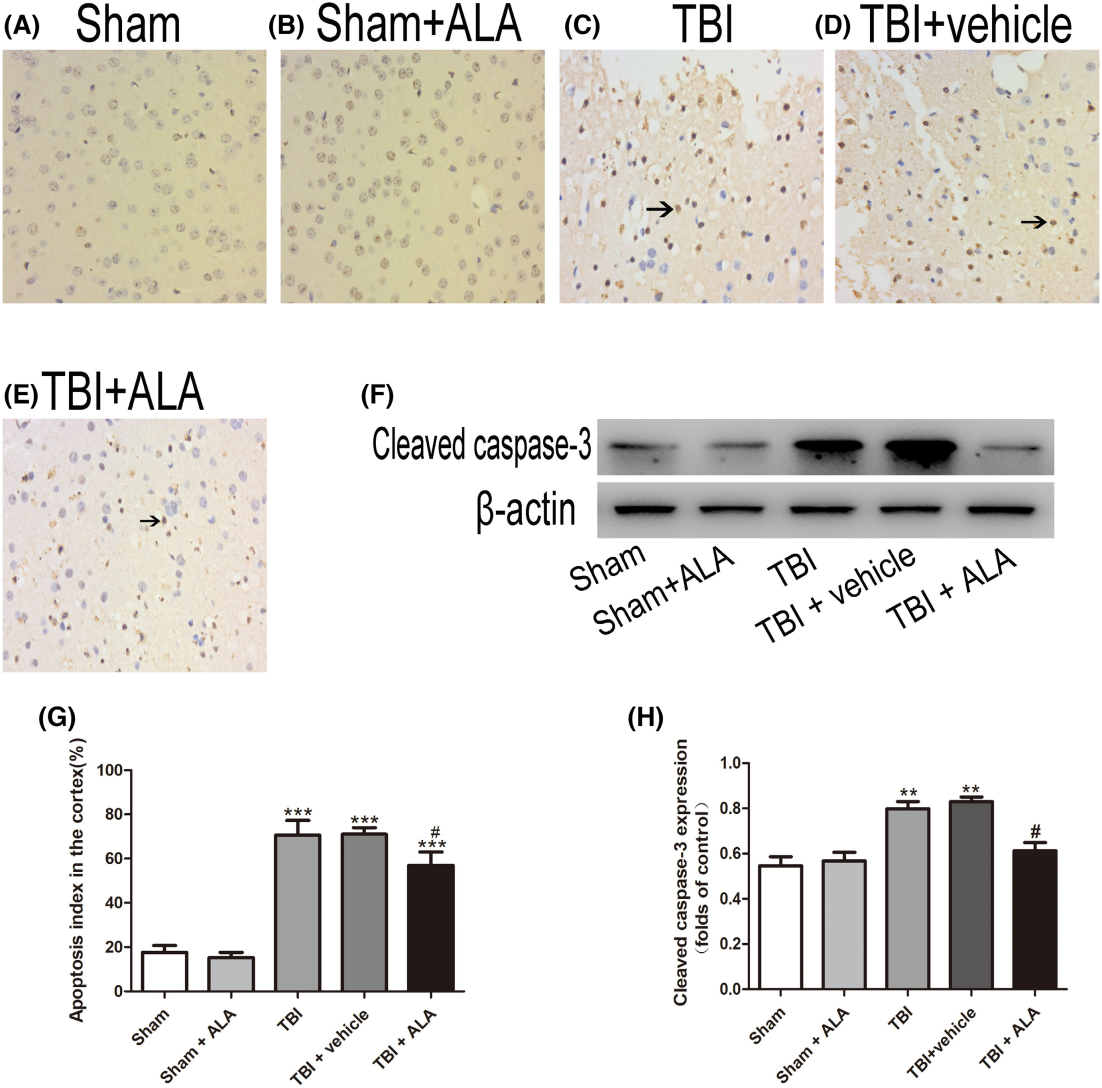
Apoptotic index was determined using TUNEL assays after TBI. ALA treatment significantly decreased the percentage of apoptotic cells after TBI. Data are presented as mean ± SD; ***p < 0.001 versus sham group; ^#^ *p* < 0.05 versus TBI + vehicle group. Black arrows: Nrf2‐positive neuron cell

## References

[1] Xia D , Zhai X , Wang H , Chen Z , Fu C , Zhu M . Alpha lipoic acid inhibits oxidative stress‐induced apoptosis by modulating of Nrf2 signalling pathway after traumatic brain injury. J Cell Mol Med. 2019;23(6):4088‐4096. 30989783 10.1111/jcmm.14296 PMC6533507

